# High clinical impact and diagnostic accuracy of EUS-guided biopsy sampling of subepithelial lesions: a prospective, comparative study

**DOI:** 10.1007/s00464-017-5808-2

**Published:** 2017-08-15

**Authors:** Per Hedenström, Hanns-Ulrich Marschall, Bengt Nilsson, Akif Demir, Björn Lindkvist, Ola Nilsson, Riadh Sadik

**Affiliations:** 1 Division of Medical Gastroenterology, Department of Internal Medicine, Sahlgrenska University Hospital, Blå Stråket 3, 413 45 Gothenburg, Sweden; 2 Department of Surgery, Sahlgrenska University Hospital, Gothenburg, Sweden; 3 Department of Clinical Pathology and Cytology, Sahlgrenska University Hospital, Gothenburg, Sweden; 4 Department of Internal Medicine and Clinical Nutrition, Institute of Medicine, Sahlgrenska Academy, University of Gothenburg, Gothenburg, Sweden

**Keywords:** Endoscopic ultrasound, Subepithelial lesion, Gastrointestinal stromal tumor, Leiomyoma, Fine needle biopsy, Clinical impact

## Abstract

**Background:**

In a tertiary center setting we aimed to study the diagnostic accuracy and clinical impact of EUS-guided biopsy sampling (EUS-FNB) with a reverse bevel needle compared with that of fine needle aspiration (EUS-FNA) in the work-up of subepithelial lesions (SEL).

**Methods:**

All patients presenting with SELs referred for EUS-guided sampling were prospectively included in 2012–2015. After randomization of the first pass modality, dual sampling with both EUS-FNB and EUS-FNA was performed in each lesion. Outcome measures in an intention-to-diagnose analysis were the diagnostic accuracy, technical failures, and adverse events. The clinical impact was measured as the performance of additional diagnostic procedures post-EUS and the rate of unwarranted resections compared with a reference cohort of SELs sampled in the same institution 2006–2011.

**Results:**

In 70 dual sampling procedures of unique lesions (size: 6–220 mm) the diagnostic sensitivity for malignancy and the overall accuracy of EUS-FNB was superior to EUS-FNA compared head-to-head (90 vs 52%, and 83 vs 49%, both *p* < 0.001). The adverse event rate of EUS-FNB was low (1.2%). EUS-FNB in 2012–2015 had a positive clinical impact in comparison with the reference cohort demonstrated by less cases referred for an additional diagnostic procedure, 12/83 (14%) vs 39/73 (53%), *p* < 0.001, and fewer unwarranted resections in cases subjected to surgery, 3/48 (6%) vs 12/35 (34%), *p* = 0.001.

**Conclusions:**

EUS-FNB with a reverse bevel needle is safe and superior to EUS-FNA in providing a conclusive diagnosis of subepithelial lesions. This biopsy sampling approach facilitates a rational clinical management and accurate treatment.

**Electronic supplementary material:**

The online version of this article (doi:10.1007/s00464-017-5808-2) contains supplementary material, which is available to authorized users.

Subepithelial lesions (SEL) is a term referring to lesions located under the mucosa and originating from the wall itself or from outside the wall [1–3]. SEL provides no indication of the entity behind the lesion. A definitive diagnosis would allow a rational clinical management [2, 4].

SELs include a wide spectrum of lesions ranging from highly malignant conditions with need for a rapid management, such as high risk gastrointestinal stromal tumors (GIST) and leiomyosarcomas, to entirely benign lesions like a heterotopic pancreas [5].

The expression SEL is used as an umbrella term since the diagnostic work-up of the entities behind these lesions has been challenging. Routine gastroscopy with forceps biopsy is of limited value and not adequate in diagnosing SELs [4, 6]. Endoscopic ultrasound (EUS) without lesion sampling is also insufficient in establishing the diagnosis [6–9]. That is, in most SELs a microscopic assessment of cells or tissue is required, not least in solid, hypoechoic lesions [10]. In numerous tumors, such as GIST, leiomyoma, and schwannoma, a definitive diagnosis cannot be obtained without immunostaining of cytology (ICC) or histology (IHC) specimens [2].

The diagnostic accuracy of EUS-guided fine needle aspiration (EUS-FNA) in SEL has been reported poor [3], especially in lesions <2 cm [11]. The tru-cut biopsy needle aimed for histology (EUS-TCB, Quick-core, Cook Medical) has been associated with a high frequency of technical failures [12, 13], low yield, and non-superior diagnostic accuracy compared with EUS-FNA [14, 15].

Hence, at present endoscopists and surgeons face SELs without a firm diagnosis after endoscopy and EUS in as much every second patient [14]. Consequently, the clinical and surgical management of these lesions can be challenging and moderately substantiated. In truly benign lesions, there is a substantial risk of unwarranted resections leading to patient morbidity or unmotivated follow-up. In addition, the health service will be affected by high and unnecessary costs. In malignant lesions, there is a risk of delayed therapy.

The general purpose of this work was to obtain a conclusive pretreatment diagnosis of lesions presenting as SELs by performing EUS-guided tissue acquisition and by engaging dedicated experts within the fields of endoscopy, pathology, and surgery.

The specific primary aim of this study was to compare the diagnostic accuracy and safety of EUS-guided reverse bevel needle biopsy sampling (EUS-FNB) with that of EUS-FNA in all malignant and benign subepithelial lesions, irrespective of size and origin. The secondary aim was to assess the impact of EUS-FNB on the clinical and surgical management of SELs.

## Materials and methods

### Study design and study population

All patients aged >18 years with a lesion presenting as a SEL and referred for a diagnostic EUS to the Sahlgrenska University Hospital 2012–2015 (the tertiary EUS-center of West Sweden, population: 1.9 million) were eligible for inclusion in this prospective, comparative study. Completely solid lesions and solid lesions with some cystic transformation, i.e., tumor necrosis, were accepted for inclusion. Completely cystic lesions were not sampled and were excluded from further analysis. Small lesion size was not an exclusion criterion.

All patients fulfilling the inclusion criteria were consecutively enrolled as study subjects and were sampled with dual needles (EUS-FNA and EUS-FNB) as discussed below. The only exclusion criteria for dual sampling with EUS-FNA and EUS-FNB was a sampling route interfered by large vessels when targeting lesions <20 mm (EUS-FNA only performed, *n* = 3).

This project was approved by the Regional Ethical Review Board of West Sweden. Written informed consent was obtained. The STARD protocol was applied throughout the study. This study was registered in the ClinicalTrials.gov database (NCT02360839).

### EUS—examination and the acquisition of cells and tissue

All study patients were examined by EUS under conscious sedation performed by either of three experienced endosonographers (RS/HUM/PH). A linear echoendoscope [Pentax EG3870UTK (Tokyo, Japan)] and an ultrasound processor (Hitachi HI VISON Ascendus) were used. The lesions examined were characterized and classified upon size, location, origin, echogenicity, and vascularization. Finally the optimal position for sampling was determined.

**Fig. 1:**
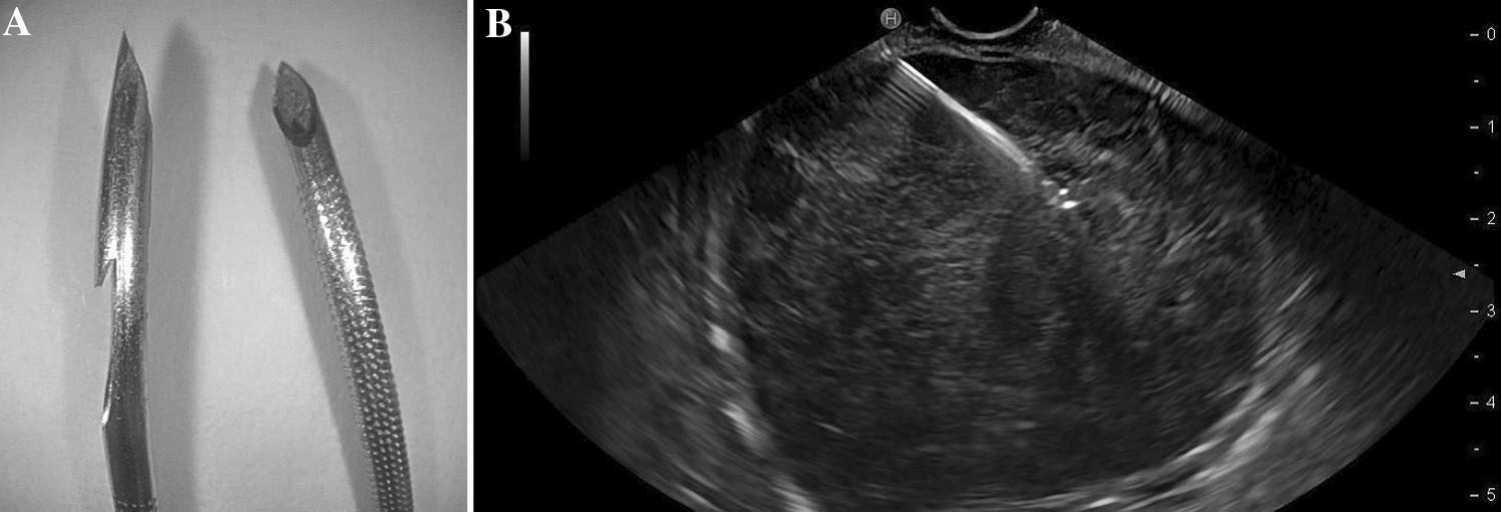
Left image (**A**) a close-up photo showing the tip of the 22 gauge FNB-needle with the side fenestrated, reverse bevel design (*left*) and the tip of a standard 25 gauge FNA-needle (*right*). The photo was captured with a Fujifilm HD Video Endoscope EG760Z (Tokyo, Japan). Right image (**B**) the endosonography image of a 6 cm hypoechoic gastrointestinal stromal tumor situated in the gastric body during EUS-guided sampling with a 22 gauge reverse bevel FNB-needle

To accustom the study endosonographers to the reverse bevel FNB-needle, the first ten patients were punctured with EUS-FNB only during a short run-in phase. Then, the sampling was performed according to the pre-defined study protocol with both EUS-FNA for cytology [EUS-FNA with a 22 or a 25 gauge needle (Olympus, Aomori, Japan/Boston Scientific, Spencer, USA/Wilson-Cook Medical, Limerick, Ireland)] and with EUS-FNB for histology (22 or 19 gauge, Wilson-Cook Medical) on each individual lesion, Fig. 1. It was left to the discretion of the endosonographer to determine the appropriate needle size in each case. By blocks of four and by using sealed envelopes the patients were randomized to first pass with FNA or FNB. Further passes were performed by alternating the needles. A technical failure was defined as the non-ability to pass the needle through the instrument or to insert the needle into the target lesion.

In both EUS-FNA and EUS-FNB, the needle tip was placed, if possible, in a non-necrotic part of the tumor. During 5–15 s and with suction applied, each needle was moved in different directions to avoid traumatized tissue, so called “fanning” [16], Fig. 1.

The first FNA-sample was smeared on to a glass, air-dried, and stained with Giemsa for rapid on-site cytology assessment (ROSE) by a cytotechnician. Residual FNA-material was put into saline or into a methanol-based solution (ThinPrep^®^, Hologic, Marlborough, USA). The access to a cytotechnician is limited in our institution, so in procedures when ROSE was not performed the complete FNA-yield was put directly into ThinPrep^®^ by the endoscopy assistant. When available, the cytotechnican also performed a gross examination of the first FNB-core, which was put into formalin. In the absence of ROSE, the gross examination was performed by the endosonographer.

The sampling procedure was continued until the yield was regarded satisfactory by the cytotechnician, i.e., no fixed number of passes was performed. For the sake of patient safety we did not exceed six passes, both modalities counted. Failures and adverse events were recorded.

### Cytopathology and histopathology

The laboratory processing of the FNA- and FNB-samples including immunohistochemistry is described in detail in Supplementary Materials.

### Clinical follow-up and reference standard of the final diagnosis

The medical history of the study subjects and the diagnostic work-up before and after the EUS was recorded. The referring clinician was responsible for the decision on surgical resection. The study subjects were monitored until the final diagnosis was established or until death. Adverse events occurring during the first 30 days post-EUS were recorded.

Based on the final diagnosis, each lesion was assigned as a *malignant neoplastic lesion*, a *benign neoplastic lesion*, or a *non*-*neoplastic lesion* [9]. A lesion was denoted “suspicious for” if no definite diagnosis was established during follow-up.

In patients subjected to surgery, the resected specimen was used as the reference standard. In not resected cases, a conclusive pathology report of the EUS-sampling itself or of an alternative sampling modality was accepted. If a tissue-based final diagnosis was not obtained, the clinical diagnosis at a minimum of 12 months follow-up was used.

Based on the pathology report, the FNA-samples and the FNB-samples were classified as diagnostic or non-diagnostic. In *neoplastic lesions*, both a representative yield and a lesion specific immunostaining profile were required to consider a sample as diagnostic [2]. The samples without a representative yield or with inconclusive immunostaining were considered non-diagnostic. In lipomas and malignancies of mucosal origin no immunochemistry was required if the cytomorphology was diagnostic per se. In *non*-*neoplastic lesions*, the EUS-samples were classified as diagnostic if containing benign, representative cells. Otherwise they were classified as non-diagnostic.

### Measurement of the clinical impact

To analyze the clinical impact of EUS-FNB we used as comparison the cohort of all cases with SEL undergoing EUS-guided sampling at our center in 2006–2011 (*the comparison cohort*). Routine EUS-FNA or EUS-TCB was performed in these cases. Else, the EUS-procedure, the laboratory processing, and the follow-up were comparable to the study cases.

In each case, we recorded any additional, diagnostic procedure post-EUS and any unwarranted surgical resection. We considered a surgical resection unwarranted if the subsequent pathology report demonstrated a diagnosis that could have been managed conservatively.

### Outcomes

The primary outcome of this study was the diagnostic accuracy of EUS-guided sampling including the number of technical failures and adverse events related to sampling.

The secondary outcome was the clinical impact of EUS-FNB measured as the diagnostic success of FNB-sampling in previous non-diagnostic FNA-cases, the performance of an additional diagnostic procedure post-EUS, and the performance of an unwarranted surgical resection.

### Statistical analysis

In a two-tailed sample size calculation for paired samples with dichotomous outcome we aimed to detect a difference in accuracy of 30% at dual sampling comparing EUS-FNA and EUS-FNB. The type I-error (α) was set at 0.05 and the statistical power at 80% (1 − β). A total sample size of 59 cases was returned. The lesion characteristics and technical procedures were compared using Fischer’s exact test (unpaired, binary data), Student’s *t* test, and Mann–Whitney *U*-test (unpaired, continuous data). The diagnostic accuracy and the clinical impact of sampling were compared between groups using Fischer’s exact test and McNemar’s test (paired, binary data).

An intention-to-diagnose analysis, including cases without yield and technical failures, was used to calculate and present the data [17]. When applicable the 95% confidence intervals (CI 95) were calculated. The statistical significance level was set at *p* < 0.05. SPSS Statistics version 22.0 (IBM Corporation, Chicago, IL, USA) was used.

## Results

**Fig. 2:**
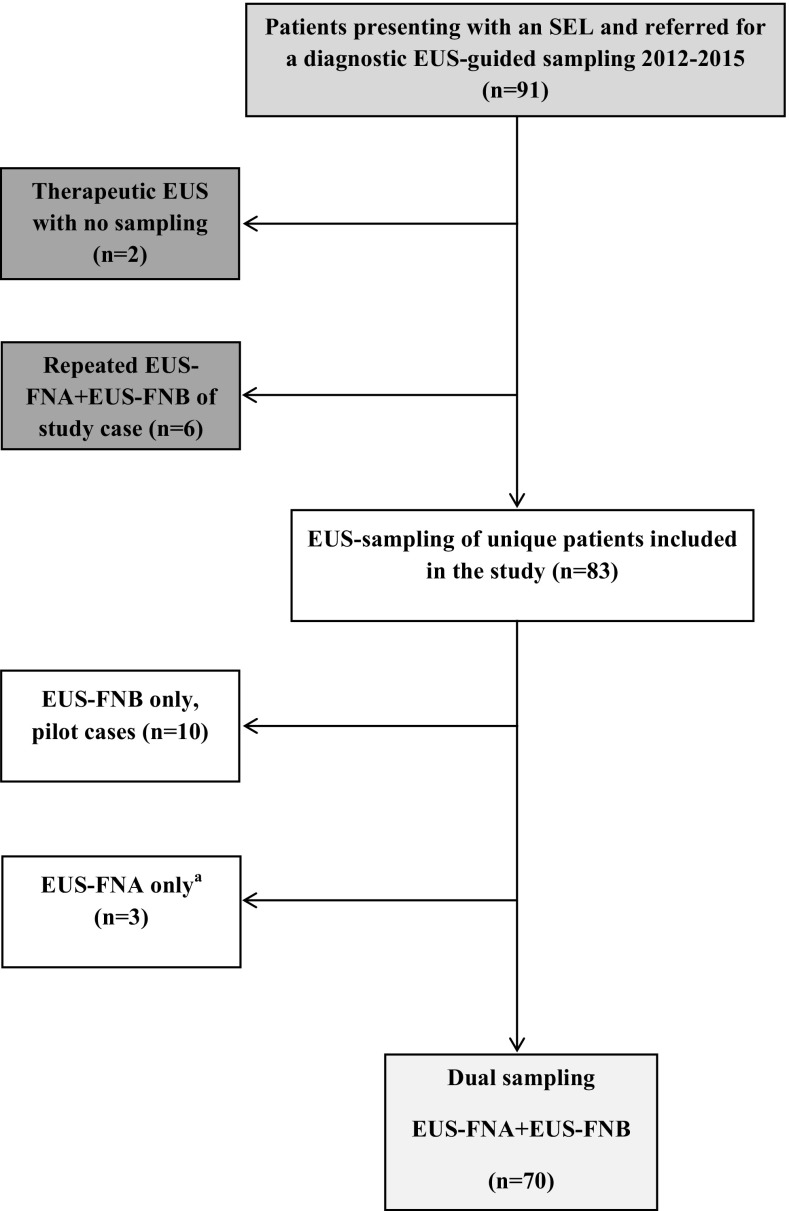
Flow chart of the study enrollment process of patients presenting with a SEL and included in the study 2012–2015 at the Sahlgrenska University Hospital endoscopy unit. *Superscript a* indicates the three small lesions (<20 mm) in which EUS-FNA only was performed due to interfering large vessels in the sampling routes

The study enrollment process is outlined in Fig. 2. During the study time frame 2012–2015 a total of 89 EUS-guided sampling procedures were performed in 83 unique study subjects [median age: 68 years (range 28–92), women: *n* = 42 (51%)], Table 1. The reference standard was histopathology [surgical resection (*n* = 48), non-EUS-sampling (*n* = 9)] in 57/83 (69%) of the cases.

**Table 1:** The study base-line characteristics

	Study cohort (2012–2015)	Comparison cohort (2006–2011)
Patient characteristics		
Number of patients, *n*	83	73
Patient age, median (range)	68 (28–92)	65 (21–89)
Patient gender, m/f	41/42	35/38
Lesion characteristics		
Neoplastic lesions—malignant, *n* (%)	63 (77)	38 (52)
Neoplastic lesions—benign, *n* (%)	13 (15)	16 (22)
Non-neoplastic lesions, *n* (%)	7 (9)	19 (26)
Location, *n*, esophagus	9	13
Stomach (fundus/body/antrum)	66 (22/36/8)	52 (12/25/15)
Duodenum	8	8
Position^a^, *n* (intramural/extramural)	72/11	62/11
Size, mm, median (range)	30 (6–220)	26 (6–220)
Echoappearance, *n*, (solid/semisolid^b^)	67/16	58/15
EUS-sampling		
FNA-needle, *n*, (22G/25G)	43/30	35/31
FNA-passes, *n*, median (range)	3 (1–4)	3 (1–6)
Rapid on-site cytology (ROSE), *n*/*n*_tot_ (%)	47/73 (64)	36/66 (55)
FNB-needle, *n* (19G/22G)	13/67	–
FNB-passes, *n*, median (range)	2 (1–4)	–
TCB^c^-needle, *n* (19G)	–	13
TCB-passes, *n*, median (range)	–	1 (1–5)
Follow-up		
Reference (histopathology/clinical course), *n* (%)	57 (69)/26 (31)	48 (66)/25 (34)
Time frame EUS–surgical resection, months, median (range)	3 (1–17)	6 (1–44)
Follow-up time^d^, months, median (range)	28 (2–59)	48 (1–91)
Survival—cases alive^e^, *n*/*n*_tot_ (%)	72/83 (87)	46/73 (63)

^a^Based on the appearance at EUS

^b^A semisolid lesion was defined as a lesion with anechoic areas, i.e., signs of necrosis

^c^TCB = tru-cut biopsy needle

^d^As by December 2016; patients not deceased were monitored for a minimum of 12 months

^e^Recorded as by December 2016

### The primary outcome

A spectrum of 21 diagnostic entities was sampled during the four study years, Table in Supplementary Material. EUS-FNB was diagnostic in 9/10 (90%) of the single EUS-FNB pilot cases. The accuracy of EUS-FNB was superior to that of EUS-FNA in the 70 cases undergoing dual sampling EUS-FNA and EUS-FNB, Table 2. No analyzed factor had significant impact on the sensitivity of EUS-FNB, Table 3. Regarding the sensitivity of EUS-FNA, three or more FNA-passes and the performance of ROSE had a positive impact, while there was a trend that an extramural lesion origin had a negative impact, Table 3.

**Table 2:** The diagnostic accuracy of sampling

Dual sampling EUS-FNA and EUS-FNB of unique study cases (2012–2015)			
Outcome parameter	EUS-FNB, % (CI 95^a^)	EUS-FNA, % (CI 95)	*p* value
Overall accuracy (*n* = 70)	83 (74–92)	49 (37–51)	<0.001
Sensitivity (*n* = 65)	83 (74–92)	48 (36–60)	<0.001
Malignant neoplastic lesions (*n* = 52)	90 (82–98)	52 (38–66)	<0.001
Benign neoplastic lesions (*n* = 13)	54 (27–81)	31 (6–56)	0.25
Specificity (*n* = 5)	80 (NC^b^)	60 (NC)	1.0

^a^The 95% confidence interval

^b^Non-countable due to low number of patients

**Table 3:** Factors influencing the sensitivity of sampling

	EUS-FNA		EUS-FNB	
	Diagnostic sample *n*/*n*_tot_ (%)	*p* value	Diagnostic sample *n*/*n*_tot_ (%)	*p* value
Needle size		1.0		0.19
25 gauge	11/23 (48)		–	
22 gauge	20/42 (48)		45/55 (82)	
19 gauge	–		9/10 (90)	
Sampling order		0.62		0.75
FNA first, FNB second	16/31 (52)		25/31 (81)	
FNB first, FNA second	15/34 (44)		29/34 (85)	
Needle passes		0.02		1.0
3 passes or more	25/42 (59)		18/22 (82)	
1–2 passes	6/23 (26)		36/43 (84)	
On-site cytopathology		0.01		p = 0.31
ROSE	26/44 (59)		38/44 (86)	
non-ROSE	5/21 (24)		16/21 (76)	
Tumor size		0.55		1.0
>20 mm	23/51 (45)		42/51 (82)	
≤20 mm	8/14 (57)		12/14 (86)	
Tumor type		1.0		0.35
Solid	26/55 (47)		47/55 (85)	
Semisolid (necrotic)	5/10 (50)		7/10 (70)	
Tumor location^a^				
Esophagus	1/6 (17)	0.19	4/6 (67)	0.27
Stomach	28/52 (54)		44/52 (85)	
Duodenum	2/7 (29)	0.25	6/7 (86)	1.0
Tumor position		0.06		0.13
Intramural	30/57 (53)		49/57 (86)	
Extramural	1/8 (13)		5/8 (63)	

^a^The stomach was set as the reference lesion location in the statistical analysis

Analyzing only the subset of cases in which ROSE was performed and in which at least three FNA-passes were performed, EUS-FNB still had a higher sensitivity compared with EUS-FNA, 28/30 (93%) versus 20/30 (66%), *p* = 0.01.

EUS-FNA was non-diagnostic in all the lesions (*n* = 12) in which EUS-FNB was also non-diagnostic. In six non-diagnostic cases a repeated dual sampling was performed; EUS-FNB was diagnostic in 5/6 (83%) and EUS-FNA in 2/6 (33%), *p* = 0.38.

In one patient with a 50 mm tumor the 22 gauge FNB-needle was not possible to be placed in position for a transduodenal pass (FNB-technical failure rate: 1/86, 1.2%). There were no failures of EUS-FNA. In one patient with a gastric GIST there was a late local bleeding (FNB-adverse event rate: 1/86, 1.2%). The bleeding was stopped the following day by gastroscopy and the local administration of 10 mL of epinephrine.

### The secondary outcome

The base-line characteristics of *the comparison cohort* 2006–2011, including 63 unique patients, were according to Table 1. The diagnostic sensitivity for malignancy and the overall accuracy of EUS-FNA (*n* = 66) in this cohort was 46% (CI 95: 29–63) and 34% (CI 95: 22–46) respectively. The overall accuracy of EUS-TCB (*n* = 13) was 23%. The shift to EUS-guided reverse bevel FNB-sampling in 2012 resulted in a significant clinical impact:

### Success of repeated sampling

Fourteen non-diagnostic cases of *the comparison cohort* later underwent repeated EUS-guided sampling. The repeated sampling was diagnostic more often in the cases re-sampled with EUS-FNB in 2012–2015 compared with the cases re-sampled with EUS-FNA in 2006–2011, 6/6 (100%) versus 2/8 (25%), *p* = 0.01.

### The performance of additional diagnostic procedures post-EUS

The accuracy of the index-EUS-FNB in 2012–2015 resulted in the reduced performance of an additional diagnostic procedure compared with *the comparison cohort* cases, Table 4a.

**Table 4:** Additional diagnostic procedures and surgical resections

	Study cohort	Comparison cohort	*p* value
	2012–2015	2006–2011	
(a) Diagnostic procedures post-EUS			
All lesions (*n*)	83	73	
No additional diagnostic procedure performed, *n* (%)	71	34	
Additional diagnostic procedure performed, *n* (%)	12 (14)	39 (53)	<0.001
Diagnostic surgical resection or biopsy	5	19	
Repeated EUS	3	13	
Repeated EUS and diagnostic resection	–	3	
PET-CT	–	2	
Transabdominal sampling	1	1	
Diagnostic endoscopic EMR	1	1	
Bronchoscopy	1	–	
Repeated endoscopy forceps biopsy	1	–	
Malignant lesions (*n*)	63	38	
No additional diagnostic procedure performed, *n* (%)	57 (90)	21 (55)	
Additional diagnostic procedure performed, *n* (%)	6 (10)	17 (45)	<0.001
Diagnostic surgical resection or biopsy	2	9	
Repeated EUS	2	4	
Repeated EUS and diagnostic resection	–	1	
PET-CT	–	2	
Transabdominal sampling	1	1	
Diagnostic endoscopic EMR	–	–	
Bronchoscopy	–	–	
Repeated endoscopy forceps biopsy	1	–	
(b) Surgical resections			
Cases subjected to surgical resection (*n*)	48	35	
Unwarranted resections, *n* (%)	3/48 (6)	12/35 (34)	0.001
Ectopic pancreatic tissue	0	3	
Leiomyoma	0	2	
Schwannoma	1	2	
Benign inflammatory tissue or scar tissue	0	2	
Lipoma	1	1	
Ganglioneurinoma	1	1	
Glomangioma	0	1	

### The performance of unwarranted resections

There were few unwarranted resections performed among the resected study cases compared with the resected cases of the *comparison cohort*, Table 4b.

## Discussion

This work performed in a large tertiary center over several years enabled us to unveil a wide spectrum of diagnostic entities hidden behind the gastrointestinal mucosa. We have shown that performing EUS-guided reverse bevel biopsy sampling is safe and accurate and opens up for a dogma shift in the management of subepithelial lesions, which should be first treated after a firm diagnosis is reached. The high diagnostic accuracy could also be translated into a clear clinical benefit demonstrated by the reduced number of both additional diagnostic procedures and unwarranted resections. In contrast, the tru-cut biopsy needle (EUS-TCB) previously used had a limited clinical impact [18].

Surgeons and oncologists are dependent on a reliable diagnosis before the initiation of therapy [19]. To personalize the management of the wide spectrum of lesions that constitute SELs, it is important to firmly characterize each lesion and relate the diagnosis to the general health of the patient. The data presented in this study strongly support a description of lesions based on the true entity and not on the appearance of the initial presentation.

A recent review on the accuracy of EUS-FNB included nine publications and stressed the need for studies focusing on subepithelial lesions [20]. The authors identified only two [21, 22] studies including SELs. In the first of these studies, 22 patients with SEL were randomized to EUS-FNB (*n* = 12) or to EUS-FNA without ROSE (*n* = 10) [21]. Excluding lesions <20 mm, the diagnostic accuracy of EUS-FNA was 20% and the accuracy of EUS-FNB was 75%. In the second study, including eighteen patients, only two gastric masses were punctured with EUS-FNB [22].

Several studies on SELs have a retrospective design [2, 23, 24]. Many studies present a diagnostic accuracy of EUS-FNA in the range 40–62% supporting the fact that EUS-FNA of SELs is non-diagnostic in around half of the cases like in our study [2, 3, 14, 24]. In a well-designed, prospective study published in 2010, ROSE was performed by a cytopathologist and the yield of EUS-FNA was reported 70% [14]. However, the diagnostic accuracy of EUS-FNA including conclusive immunostaining only reached 52%, a number equivalent to our study. In a very recent publication on SELs by El Chafic and collaborators, a firm diagnosis including conclusive immunostaining was reached by EUS-FNA including ROSE in only 53% of the cases [23].

In the presented study, ROSE increased the accuracy of EUS-FNA, which however did not reach the accuracy of EUS-FNB. We noticed a trend that the sensitivity of EUS-FNA was lower in extramural lesions compared with intramural lesions, but no such trend existed regarding EUS-FNB, Table 3. An explanation could be that extramural lesions in general are more mobile during sampling when the tissue is attached to the gastrointestinal wall. As it seems, the biopsy needle used in this study might be less affected by this circumstance.

In the literature it is common to present the sample adequacy and yield as separate outcomes and exclude cases without material in the calculation of the diagnostic accuracy. In this study we applied a conservative and clinical approach when classifying the samples. All punctured cases were included and analyzed in an intention-to-diagnose analysis. The reason for that is that an accurate clinical decision should be based on a definitive diagnosis [25], which in our study was obtained in 90% of the malignant lesions after the index-EUS-FNB.

In this study we did not exclude small lesions. This approach is supported by a study showing that GISTs less than 2 cm in diameter can metastasize [10] and by the recommendation to resect all non-metastatic GISTs to reduce the risk of recurrence [26]. On the other hand, leiomyomas and schwannomas should not be resected due to a low malignant potential [27]. This fact stresses the importance of immunostaining of samples to discriminate different tumors from each other.

We recorded a low technical failure rate of reverse bevel EUS-FNB giving further support to avoid EUS-TCB [12]. Except in one case, we could perform EUS-FNB for transduodenal punctures. Such difficulties have been described in some publications [28, 29], but not in others [21, 30].

In accordance with the available literature we recorded few adverse events using the reverse bevel FNB-needle [21, 28, 29]. The dual sampling approach FNA + FNB did not seem to have any additional negative impact on the patient safety.

There are few publications analyzing the clinical impact of EUS-guided sampling in SELs [18]. The issue is important to clinicians and surgeons deciding on treatment and follow-up. Additional diagnostic procedures and unmotivated follow-up can have a negative impact on patient well-being. The performance of EUS-FNB in SELs probably would make the diagnostic work-up effective and save costs for the health care providers.

This work included all SELs referred for a EUS-guided sampling during the time frame of 4 years. The extensive sampling approach with dual EUS-FNA and EUS-FNB in the same SEL enabled us to compare the modalities head-to-head. Furthermore, the presented comparison with data of a reference cohort of SELs at the same institution allowed us to analyze the clinical impact of reverse bevel EUS-FNB. The use of a study specific cytopathologist and pathologist minimized the sample assessment variability. Finally, we applied strict criteria to classify the samples as diagnostic, which is relevant in the clinical context [2].

A weakness of the presented work is that it is a single-center study. The large volume of patients and the pathology expertise familiar with EUS-samples might not be available in all centers. Another weakness of the study was that the cytotechnician was not available for ROSE in all the study FNA-samplings. If that would have been the case, the diagnostic accuracy of EUS-FNA could have somewhat higher, but most probably still inferior to EUS-FNB.

In conclusion, this prospective study shows that a wide spectrum of diagnostic entities can present as subepithelial lesions and that EUS-guided reverse bevel biopsy sampling is a safe and accurate diagnostic tool. The FNB-sampling approach opens the door for a more personalized management of patients affected by subepithelial lesions.

## Electronic supplementary material

Below is the link to the electronic supplementary material.

Supplementary material 1 (DOCX 21 kb)

## Abbreviations

EUS: Endoscopic ultrasound
FNA: Fine needle aspiration
TCB: Tru-cut biopsy sampling
FNB: Fine needle biopsy sampling
GIST: Gastrointestinal stromal tumor
ROSE: Rapid on-site evaluation
ICC: Immunocytochemistry
IHC: Immunohistochemistry
