# Supplementary material for: High clinical impact and diagnostic accuracy of EUS-guided biopsy sampling of subepithelial lesions: a prospective, comparative study
Source: Surg Endosc. 2017 Aug 15;32(3):1304–13. doi: 10.1007/s00464-017-5808-2 (PMC5807503; doi:10.1007/s00464-017-5808-2)
Supplement: Supplementary file 1 — Supplementary material 1 (DOCX 21 kb) [file 464_2017_5808_MOESM1_ESM.docx]

**Material and methods**

*Cytopathology and histopathology*

The FNA-material stored in ThinPrep^®^ was used for the preparation of cell-blocks by the use of an automatized system (Cellient^™^, Hologic) according to the manufacturer´s instructions. The cell-blocks were paraffin-embedded, cut in thin sections, and placed on slides. Prior to immunostaining the slides were fixed in acetone at -20°C for 10 minutes, then treated in a microwave oven (at 700W for 7 minutes and 300W for 15 minutes) with a Dako Target Retrieval Solution (citrate pH 6, S2031), then cooled to room temperature and rinsed with deionized water.

The FNB-core biopsy samples were formalin-fixed and paraffin-embedded (FFPE). Sections (3‒4 µm) were placed on positively charged glass slides and treated in Dako PT-Link using EnVision™ FLEX Target Retrieval Solution (TRS High).

Subsequently the samples were further analyzed by immunocytochemistry and immunohistochemistry, respectively. No fixed panel of antibodies was used, but instead the cytopathologist/pathologist selected primary, monoclonal antibodies based on the preliminary results of Giemsa or hematoxylin-eosin-staining and the suspicious diagnosis in each individual case. Most frequently used antibodies were c-KIT, DOG-1, CD34, smooth muscle actin, desmin, S-100, synaptophysin, chromogranin A, and cytokeratin. Immunostaining was performed in a Dako Autostainer Link using EnVision™ FLEX according to the manufacturer’s instructions (DakoCytomation). Positive and negative controls were included in each run.

Any relevant clinical information and the lesion endosonography characteristics were supplied to the study cytopathologist (AD) and pathologist (ON); responsible for the sample assessment.

**Supplementary Table**

|  | **Cases, n (%)** | **Location, n** |
| --- | --- | --- |
|  |  | (esophagus/stomach/duodenum) |
| **Unique study lesions (2012-2015)** | **83 (100)** | **9/65/9** |
|  |  |  |
| **Neoplastic lesions - malignant** | **63 (77)** | **3/51/9** |
| GIST | 47 (57) | 1/42/4 |
| GIST, *suspicious for* | 2 (2) | 0/2/0 |
| Malignant sarcoma | 3 (4) | 0/2/1 |
| Gastric adenocarcinoma | 2 (2) | 0/2/0 |
| Duodenal gastrinoma | 1 (1) | 0/0/1 |
| Breast cancer metastasis | 1 (1) | 0/1/0 |
| Duodenal adenocarcinoma | 1 (1) | 0/0/1 |
| Adenocarcinoma unclear primary | 1 (1) | 0/0/1 |
| Lymphoma | 1 (1) | 0/0/1 |
| SCLC^a^ | 1 (1) | 1/0/0 |
| Metastasis thyroid cancer | 1 (1) | 1/0/0 |
| Metastasis melanoma | 1 (1) | 0/1/0 |
| MPNST^b^ | 1 (1) | 0/1/0 |
|  |  |  |
| **Neoplastic lesions - benign** | **13 (15)** | **4/9/0** |
| Leiomyoma | 7 (8) | 3/4/0 |
| Leiomyoma, *suspicious for* | 1 (1) | 1/0/0 |
| Schwannoma | 1 (1) | 0/1/0 |
| Lipoma | 1 (1) | 0/1/0 |
| ECL-carcinoid | 2 (2) | 0/2/0 |
| Ganglioneurinoma | 1 (1) | 0/1/0 |
|  |  |  |
| **Non-neoplastic lesions** | **7 (9)** | **2/5/0** |
| Scar or inflammatory tissue | 4 (5) | 2/2/0 |
| Heterotopic pancreas | 1 (1) | 0/1/0 |
| Heterotopic splenic tissue | 1 (1) | 0/1/0 |
| Heterotopic liver tissue | 1 (1) | 0/1/0 |
|  |  |  |

^a^) SCLC = small cell lung cancer ^b^) MPNST = malignant peripheral nerve sheet tumor
